# Nationwide epidemiological characteristics of chronic fatigue syndrome in South Korea

**DOI:** 10.1186/s12967-021-03170-0

**Published:** 2021-12-07

**Authors:** Eun-Jin Lim, Jin-Seok Lee, Eun-Jung Lee, Seok-Ju Jeong, Ho-Young Park, Yo-Chan Ahn, Chang-Gue Son

**Affiliations:** 1 Institute of Bioscience & Integrative Medicine, College of Korean Medicine, Daejeon Oriental Hospital of Daejeon University, Daedeok-Daero 176, Seo-Gu, Daejeon, 35235 Republic of Korea; 2 Department of Integrative Medicine, Graduate School of Integrative Medicine, CHA University, 120 Haeryong-Ro, Kyeong-Gi, Pocheon, 11160 Republic of Korea; 3 Department of Korean Rehabilitation Medicine, College of Korean Medicine, Daejeon University, Daedeok-Daero 176, Seo-Gu, Daejeon, 35235 Republic of Korea; 4 Health Insurance Review & Assessment Service, Dunsanbuk-ro 121, Seo-Gu, Daejeon, 35236 Republic of Korea; 5 Department of Health Service Management, Daejeon University, 96-3 Yongun-Dong, Dong-Gu, 300-716, Daejeon, 34520 Republic of Korea

**Keywords:** Chronic fatigue syndrome, CFS, ME/CFS, Incidence, Prevalence, South Korea, epidemiology

## Abstract

**Background:**

Chronic fatigue syndrome (CFS) is a long-term disabling illness accompanied by medically unexplained fatigue. This study aimed to explore the epidemiological characteristics of CFS in South Korea.

**Methods:**

Using the nationwide medical records provided by the Korean Health Insurance Review & Assessment Service (HIRA), we analyzed the entire dataset for CFS patients diagnosed by physicians in South Korea from January 2010 to December 2020.

**Results:**

The annual mean incidence of CFS was estimated to be 44.71 ± 6.10 cases per 100,000 individuals [95% CI: 40.57, 48.76], and the prevalence rate was 57.70 ± 12.20 cases per 100,000 individuals [95% CI: 49.40, 65.79]. These two rates increased by 1.53- and 1.94-fold from 2010 to 2020, respectively, and showed an increasing trend with aging and an approximately 1.5-fold female predominance.

**Conclusions:**

This study is the first to report the nationwide epidemiological features of CFS, which reflects the clinical reality of CFS diagnosis and care in South Korea. This study will be a valuable reference for studies of CFS in the future.

## Background

Uncontrolled chronic fatigue substantially impairs health-related quality of life (QoL), especially in cases of medically unexplained fatigue compared to explained chronic fatigue [1]. Chronic fatigue syndrome (CFS, also called 'myalgic encephalomyelitis' (ME)) is the most debilitating form of medically unexplained fatigue, which leads to a house- or bed-bound state in 25 to 29% of patients [2] and presents a sevenfold higher risk of suicide than healthy subjects [3]. The etiology of CFS is uncertain, and to date, there has been a failure to establish concrete pathophysiology, objective diagnostics, or therapeutics [4].

The prevalence of chronic fatigue is approximately 10% in the general population [5], while the CFS prevalence rate is estimated to be 1%, although this varies depending on gender, study population, ethnicity and case definition [6]. Among those factors, case definition and diagnostic methods particularly affect the prevalence rate of CFS; for example, rates of 0.9% by CDC-1994 *vs.* 0.2% by Holmes' definitions and 1.1% via interviews *vs.* 0.1% via physician diagnosis were reported [7]. Some studies reported a 1.5- to 2.0-fold female predominance attributable to sex-hormonal responses and two age peaks at 10–19 and 30–39 years [8, 9].

CFS is now considered a serious health problem in the form of a complex multisystem neurological disorder, and the Institute of Medicine (IOM) emphasized the need for national medical and scientific efforts in CFS research [10]. To date, CFS-related studies have been mainly conducted in the USA and UK. To date, five studies have assessed the prevalence of CFS in South Korea; however, these studies were conducted using relatively small populations and have not been updated since 2008 [11]. Defining an accurate prevalence rate and its related factors are essential for exploring the pathophysiological basis of CFS [12]. Meanwhile, there is still a tendency for physicians to hesitate or refuse to diagnose an individual with CFS due to a lack of knowledge or understanding of CFS [13–15].

Therefore, this study aimed to explore the nationwide clinic-based comprehensive epidemiological features of CFS in South Korea.

## Methods

### Data sources

South Korea has a national health insurance system that covers the entire population of Korea. Accurate medical information for the entire population is deposited and publicly available by the Health Insurance Review & Assessment Service (HIRA) in South Korea [HIRA]. Using the Healthcare Big Data Hub of HIRA, we extracted the entire dataset for health services to patients who had been diagnosed or treated for postviral fatigue syndrome (PVFS; this is the same as chronic fatigue syndrome, ICD10-G93.3) during the past 11 years (from January 1st, 2010, to December 31st, 2020).

### Data analysis

We analyzed the epidemiological features of CFS among the entire South Korean population using HIRA-derived datasets to address the following questions: absolute frequency of CFS based on the year, sex and age and in relation to whole South Korean population, treatment periods associated with CFS after 1st diagnosis on 2011, and any specific disease profile present before the initial diagnosis with CFS. Regarding the estimation of incidence rates, we considered an event a ‘new’ diagnosis of CFS in each year, if there was no preceding diagnosis of CFS in the patient’s medical record. For the prevalence of CFS, we extracted all cases of CFS diagnosis in each year.

### Statistical analysis

The annual incidence rate and prevalence of CFS, including 95% confidence intervals, were calculated by the number of recorded diagnosis cases divided by the number in the entire general population for each corresponding year. Subsequently, the overall rate with a 95% confidence interval (95% CI) per 100,000 patients was calculated. The data for the whole population were obtained from the Korean Statistical Information Service [16]. Statistical analyses of any differences between males and females were conducted by the Mann–Whitney U test with the SPSS statistical software package version 18.0 (SPSS Inc., Chicago, IL, USA).

## Results

### Absolute number of patients newly diagnosed with CFS in each year

**Fig. 1:**
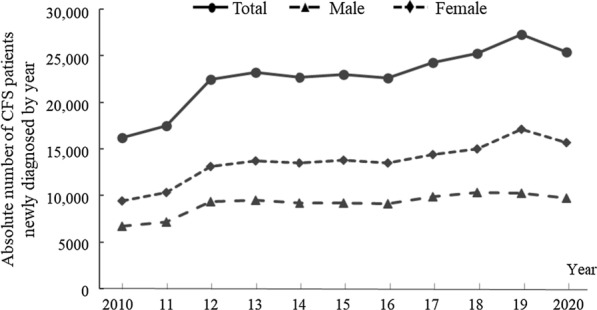
Number of patients newly diagnosed with CFS in each year. The absolute number of CFS cases from 2010 to 2020 is presented

The mean number of patients newly diagnosed with CFS in each year was 22,720 ± 3284, which showed a consistent female predominance of 1.49-fold (males 9122 ± 1152 *vs.* females 13,598 ± 2179) over 11 years. This physician diagnosis-derived annual incidence of CFS increased 1.57-fold between 2010 (16,175) and 2020 (25,403) and was slightly higher in females (1.66-fold) than in males (1.44-fold) (Fig. 1).

### Age-related features of a new CFS diagnosis

**Fig. 2:**
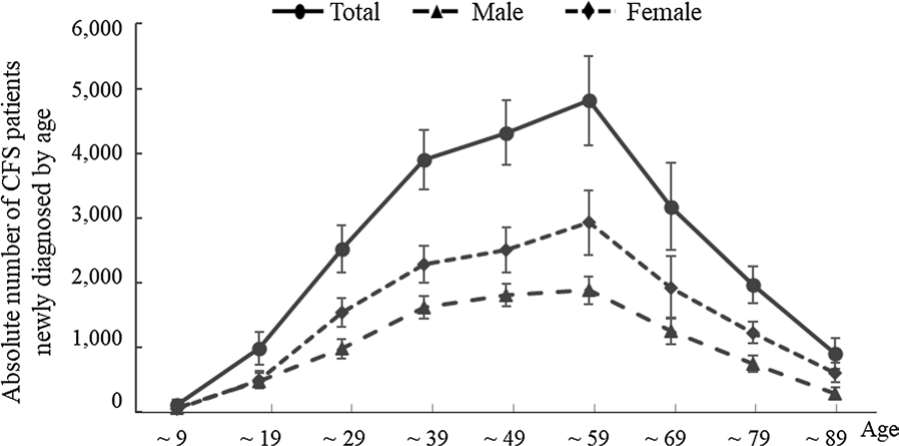
Mean number of patients newly diagnosed with CFS by age. The mean number of CFS cases by age from 2010 to 2020 is presented

Regarding the age-related frequency of CFS diagnosis, the absolute number peaked in the 50- to 59-year-old population in both males and females. Females were predominant compared to males across all age populations, with the exception of those before 10 years of age (Fig. 2).

### Relative frequency of patients newly diagnosed with CFS to the whole population

**Fig. 3:**
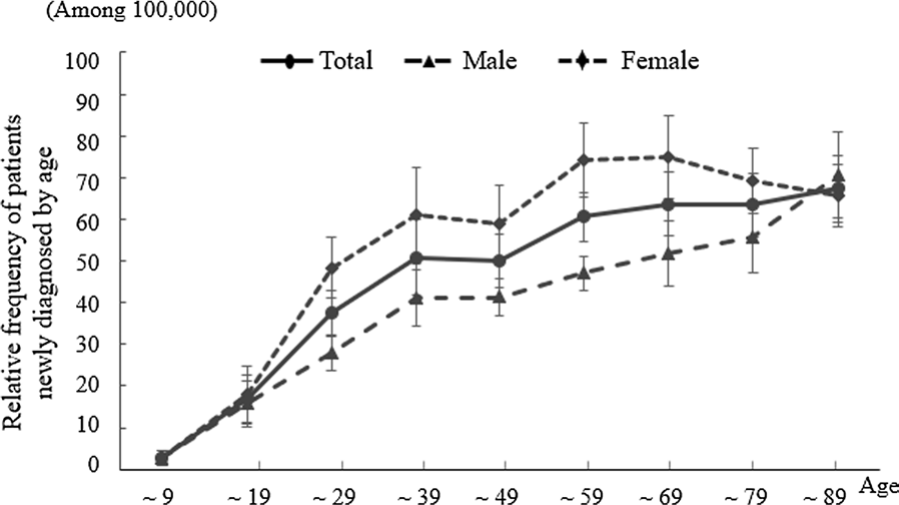
Incidence rate of CFS by age. The number of newly diagnosed CFS cases per 100,000 persons by age from 2010 to 2020 is presented

When we analyzed CFS data compared to the whole population, the average annual incidence of CFS was estimated to be 44.71 ± 6.10 cases per 100,000 individuals [95% CI: 40.57, 48.76]. There was an increase of 1.53-fold between 2010 and 2020 (from 32.43 to 49.47) (Table 1). Females (53.49 ± 8.05, 95% CI: 48.00, 58.82] were 1.49-fold more likely than males (35.93 ± 4.30, 95% CI: 33.01, 38.79] to be diagnosed, and this female-predominant pattern was consistent across the population aged 10 to 79 years old. The peak frequency in males was in the age group older than 80 years old (relative frequency of 70.7 patients), while females reached a relative frequency of 70.7 patients in the age group of 60 to 69 years old (Fig. 3).

**Table 1:** Summary of demographic features of CFS patients in South Korea

Items	Total	Male	Female	F/M ratio
Entire population	50,812,880 ± 522,785	25,389,442 ± 227,238	25,423,437 ± 295,787	–
Mean N. of new CFS patients	22,720 ± 3,284	9,122 ± 1,152	13,598 ± 2176	1.49-folds
Incidence rate (per 10^5^) (95% confidence interval) In 2010 *vs.* 2020 year (Change during 11-yrs)	44.71 ± 6.10 [40.57; 48.76] 32.43 *vs.* 49.47 (1.53-folds)	35.93 ± 4.30 [33.01; 38.79] 26.97 *vs.* 37.90 (1.41-folds)	53.49 ± 8.05 [48.00; 58.82] 37.90 *vs.* 60.98 (1.61-folds)	1.49-folds^*^ [1.43; 1.54]
Mean N. of recorded CFS patients	29,320 ± 6,456	11,747 ± 2,344	17,573 ± 4,132	1.50-folds
Prevalence rate (per 10^5^) (95% confidence interval) In 2010 *vs.* 2020 year (Change during 11-yrs)	57.70 ± 12.20 [49.40; 65.79] 36.15 *vs.* 76.10 (1.94-folds)	46.27 ± 8.88 [40.23; 52.17] 30.29 *vs.* 53.80 (1.78-folds)	69.12 ± 15.55 [58.52; 79.41] 42.03 *vs.* 86.31 (2.05-folds)	1.49-folds^*^ [1.44; 1.53]
Mean treatment duration (month)	9.13 [4.63; 13.63]	8.15 [1.73; 14.57]	9.80 [3.41; 16.19]	1.20-folds

The mean data resulted from 11-year recorded cases between 2010 and 2020. *, P < 0.001

### Treatment duration of CFS after initial diagnosis

**Fig. 4:**
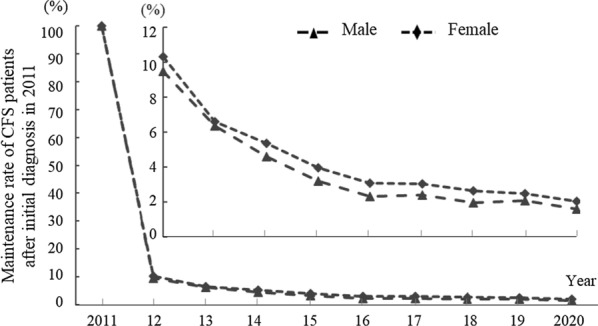
Treatment duration of CFS after being newly diagnosed in 2011. After an initial diagnosis in 2011, the percentage of CFS patients receiving treatment based on medical records through 2020 is presented. (The subfigure indicates the male and female treatment duration of CFS from 2012 to 2020)

To estimate the period during which CFS patients underwent treatment after the initial diagnosis, we analyzed individual medical records using only the patients diagnosed in 2011. In the subsequent year, only 9.97% of the patients initially diagnosed with CFS in 2011 were treated for CFS, and the rate continuously decreased to 1.86% in 2020. Based on these results, the median treatment duration for CFS seems to be approximately 9.13 months, at least in the clinic. This pattern was observed in both male and female patients, with a slightly higher rate in females than males (Fig. 4).

### Prevalence features of CFS by gender, age, and year

The mean recorded prevalence rate of CFS was 57.70 ± 12.20 cases per 100,000 individuals [95% CI: 49.40, 65.79], with a 1.49-fold female-predominant trend (69.12 ± 15.55, 95% CI: 58.52, 79.41] compared to males (46.27 ± 8.88, 95% CI: 40.23, 52.17). Over the past 11 years, this prevalence increased approximately twofold, and this increase was the same in males (1.78-fold) and females (2.05-fold) (Table 1). Similar to the annual incidence rates, the prevalence rate in males peaked at age of more than 80 years old (96.47 cases per 100,000 people) and in females in the 60- to 69-year-old range (107.90 cases per 100,000 people) (Fig. 3).

## Discussion

Signing up for the national health insurance system is mandatory for every citizen and every medical clinic in South Korea; thus, all data regarding diagnosis and treatment are deposited in the HIRA system of the Korean government. These resources are also available for beneficial public use via the Healthcare Big Data Hub of HIRA, from which we obtained the datasets for CFS. We herein produced the annual frequency of CFS diagnosis and the corresponding period of treatment by gender, age and year.

Based on the recorded diagnosis by physicians, the mean annual incidence rate of CFS in South Korea was 44.71 cases per 100,000 persons (Table 1). This annual incidence rate is higher than those reported in other studies. A previous study conducted in Wichita in the USA showed a much higher incidence rate of 180 per 100,000 person-years by the CDC 1994 case definition [17]. This study was conducted via a 1-year follow-up telephone interview and clinical examination among the general population. Another US study revealed 13.2 per 100,000 person-years using one set of regional medical records (Olmsted County in Minnesota) from 1998 to 2002 [18]. The incidence and prevalence of CFS could differ based on the applied case definitions [19]. These two studies adapted the same CDC 1994 criteria; however, they showed a large difference in CFS incidence, which might have been caused by study methods and population (survey from general population *vs*. retrospective medical record review). In a similar format as our study using the General Practice Research Database (GPRD) that includes data from participating general practices across the UK, the annual incidence was 27.0 CFS/ME/PVFS cases per 100,000 persons for the period 2001–2013 [20].

On the other hand, the prevalence of CFS is known to be approximately 1% worldwide [6]. Our previous meta-analysis using 56 datasets reported an approximately 0.89% global prevalence of CFS in the general population [7]. The present results, however, unexpectedly showed a very low prevalence, only 57.70 cases per 100,000 persons, equal to 0.057% of the general population (Table 1). This large gap might have been a result of the methods used in assessing the prevalence, namely, a questionnaire-based survey for the general population versus physician-diagnosed recorded patients. In fact, most of the studies in our previous meta-analysis used interviews, and the subgroup prevalence based on physician diagnosis (6 datasets) showed a 0.09% prevalence [7]. The early diagnosis of CFS in primary care is critically important along with tailored management; however, many medical doctors in the USA are reluctant to make a diagnosis due to the limited knowledge of the disease [21]. Moreover, a certain number of physicians tend to reject CFS as an actual physical disease, which disrupts the reliable relationship between medical providers and patients suffering from CFS [22, 23]. There remains the poor conceptual status of CFS among physicians, and South Korea is no exception, and perhaps worse, which may be a cause of South Korean CFS patients avoiding medical care [24].

Additionally, the loose definition of CFS can be another cause of the low rate of physician diagnosis. For example, the ICD-10 included ME but not CFS under PVFS (G93.3). CFS tended to be considered fatigue and miscoded as ‘fatigue syndrome’ (F48.0) or ‘malaise and fatigue’ (R53) [24, 25]. As the latest version of the ICD-11 (2019) included both ME and CFS under PVFS (8E49), awareness of the illness may be increasing [19, 26].

This would be evidenced by the result that less than 10% of CFS patients initially diagnosed with CFS in 2011 continued treatment based on medical records as CFS patients in the next year (2012), which was down to 1.86% in 2020 (Fig. 3). The median duration of CFS was reported to be approximately seven years [27], which is much longer than South Korean CFS patients were treated (approximately 9.13 months in clinics; Table 1) based on the present results. In our study, the prevalence rate of CFS was just 1.29-fold higher than its incidence rate, while a US study showed a prevalence rate 5.43-fold higher than the incidence rate [18]. If we re-estimate the prevalence rate using a 7-year median duration model, the prevalence rate of South Korean CFS would be approximately 5.1-fold higher than the incidence rate, resulting in over 0.22% of the general population. However, we anticipate that South Korean physicians are now more aware of CFS due to the increasing trends in both the incidence rate (1.53-fold) and prevalence rate (1.94-fold) (Table 1).

The female predominance, which mainly affects young adults, is a known epidemiologic feature of CFS [7, 28]. In the present study, both the incidence and prevalence rates were 1.49-fold female predominant (*p* < 0.001) (Table 1), in accordance with the meta-analysis using 56 worldwide datasets [7]. This sex-related difference in CFS incidence/prevalence is supposed to be linked primarily to sex hormones, which is supported by the difference starting at puberty, approximately 13 years of age [9, 29, 30]. Our current results also showed a higher incidence rate in males in the 9-year-old and younger subgroup (when divided into 10-year intervals), while the female-predominant pattern was observed after that point (Fig. 3). In contrast to our expectation, the total incidence rate of CFS increased with increases in age, but females peaked at 60 to 69 years old (Fig. 3). These results were somewhat dissimilar to the known facts that this illness is most common in adults between 40 and 60 years old [30]. One study using a large sample of physicians and a hospital-based database showed similar epidemiologic features, an aging-dependent increase in the total prevalence rate but a high prevalence in females between 40 and 59 years of age [31].

This study, however, contains inevitable limitations that we have to be aware of in interpreting the data. The present results for the incidence rate and prevalence rate were much lower than both other reports and our expectation. To date, 5 studies have estimated South Korean CFS prevalence, and these data-derived meta-analyses presented a rate of 0.77% (95% CI: 0.34, 1.76; a 13.5-fold increase over the present rate) along with an over twofold female predominance (1.31% in females *vs*. 0.60% in males) [11]. Among the 5 studies, 2 studies used only interviews [32–34] And 3 studies used interviews and medical tests [34–36]. All 5 studies applied CDC-1994 criteria in the CFS diagnosis process, which is the most commonly used criterion in clinics and clinical trials [7, 37]. However, we cannot verify which criteria the physicians used for diagnosis in the present dataset. We additionally examined regional difference (megacities *vs.* others) and specific profiles of disorders prior to the 1st diagnosis of CFS among the CFS patients, and no significant results were found.

The physician diagnosis-derived present data reflects the clinical reality in South Korea; however, these data might show a large difference from results from small population-based epidemiological studies. One study estimated that 84–91% of patients suffering from CFS/ME in the USA were not diagnosed with the disease by physicians [38]. There would be a possibility that CFS patients are less likely to be diagnosed by medical doctors in Korea, while they popularly use of natural remedies including ginseng product or antioxidants. In fact, many antioxidant products showed the antifatigue effects in clinical trials for chronic fatigue or CFS, and a nano-antioxidant therapy is also newly applied [39–41]. To the best of our knowledge, this study is the first to report the nationwide prevalence of CFS without any restrictions, including age.

## Conclusions

We herein characterized the nationwide epidemiological features of CFS reflecting the clinical reality using the entire physician diagnosis-derived datasets in South Korea from 2010 to 2020. The incidence rate of CFS in South Korea was estimated to be 44.71 ± 6.10 cases per 100,000 individuals [95% CI: 40.57, 48.76], and the prevalence rate was estimated to be 57.70 ± 12.20 cases per 100,000 individuals [95% CI: 49.40, 65.79]. These frequencies increased with aging and showed an approximately 1.5-fold female predominance**.** This study is the first to report the nationwide epidemiological features of CFS, which reflects the clinical reality of CFS diagnosis and care in South Korea. This study will be a valuable reference to health care providers and researchers for studies of CFS in the future.

## Data Availability

The datasets of the current study are available from the corresponding author on reasonable request.

## Abbreviations

CFS: Chronic fatigue syndrome
ME: Myalgic Encephalomyelitis
HIRA: The Korean Health Insurance Review and Assessment Service
CDC: Centers for disease control and prevention
